# ChatGPT - Reshaping medical education and clinical management

**DOI:** 10.12669/pjms.39.2.7653

**Published:** 2023

**Authors:** Rehan Ahmed Khan, Masood Jawaid, Aymen Rehan Khan, Madiha Sajjad

**Affiliations:** 1 Rehan Ahmed Khan, MBBS, FCPS, FRCS, MHPE, PhD (Medical Education), Dean Riphah Institute of Assessment, HOD & Prof. of Surgery, Islamic International Medical College, Riphah International University; 2 Masood Jawaid, MBBS, MCPS, MRCS, FCPS, MHPE, Director Medical Affairs, PharmEvo (Pvt) Ltd; 3 Aymen Rehan Khan, Final year student, Department of English, Foundation University, Rawalpindi; 4 Madiha Sajjad, MBBS, FCPS, MHPE, Prof. of Pathology, Islamic International Medical College, Riphah International University

**Keywords:** Artificial Intelligence, medical education, clinical management, ChatGPT, NLP, Education, Open AI

## Abstract

Artificial Intelligence is no more the talk of the fiction read in novels or seen in movies. It has been making inroads slowly and gradually in medical education and clinical management of patients apart from all other walks of life. Recently, chatbots particularly ChatGPT, were developed and trained, using a huge amount of textual data from the internet. This has made a significant impact on our approach in medical science. Though there are benefits of this new technology, a lot of caution is required for its use.

## BACKGROUND

Artificial Intelligence (AI) is the new genre which has been steadily taking root and is now a powerful reality in the existing world. It was based on the assumption that the process of human thought and reasoning can be replicated and mechanized. This concept emerged from a distant dream we saw in movies like ‘The Matrix’ and the ‘Terminator’ series with the likes of robots and humanoids and materialized into a strong, common day reality. We are living in the age of ‘big data’ and its actual application in a multitude of industries; technology, banking, marketing, entertainment etc., with tools like google maps, facial recognition technology, digital smart assistants like Siri and Alexa etc. to name a few. The journey continues. AI language seems to be the next big thing, and already advancing at an astonishing speed. This includes Chatbots^1^ like ChatGPT, Jasperchat, DialoGPT, Replika etc., and transcription technology like Otter.ai. ChatGPT is the most widely known of the list with an amazing ability of content generation^2^. ChatGPT is a large-scale language model developed by Open AI.

It is modeled on Generative Pre-trained Transformer (GPT), which was trained using a huge amount of textual data from the internet^2^. The purpose of ChatGPT is to generate text that mimics natural human language and can be used for multiple processing tasks such as language translation, text summarization, and dialogue systems. It can also be used to generate responses in a chat bot, answer questions, author creative stories, etc. Since it has been trained using massive amounts of text data online, the quality of text generated is similar to legitimate human language.^3^ The Generative Pre-Trained Transformer is designed to process sequential data such as natural language, and therefore allows ChatGPT to better understand the context behind sentences, how it relates to the text before it, and to generate coherent text accordingly.

### ChatGPT Role in Medical education:

Although ChatGPT has raised concerns about plagiarism and cheating^4^, it can still be utilized in several ways to improve the quality of education. Medical Education is progressing with advancements in technology, and AI such as Chat GPT, can play several helpful roles.

***1. Automated Scoring:*** ChatGPT can be effectively used to assess student papers and essays and analyze the sentence structure, vocabulary, grammar, and clarity of a paper. This feature is especially helpful for teachers and educators who are often burdened by the huge workload that comes with grading a large number of assignments.^5^,^6^

***2. Teaching Assistance:*** Another use of ChatGPT is its ability to generate exercises, quizzes and scenarios that can be used in the classroom to help practice and assess. Its ability to generate translations, explanations, and summaries can also be used to help in making complex learning material easier for students to understand.^7^,^8^

***3. Personalized Learning:*** ChatGPT can be used to create virtual tutors or assistants that can answer students’ questions as well as provide feedback on their work. Personalized study plans and learning materials can also be arranged, according to different learning styles and abilities of students; a task which may be difficult for teachers to accomplish for individual students in a classroom setting.

***4. Research Assistance:*** ChatGPT can also be utilized to assist students in their research by answering questions and providing summaries of texts. It may also be used to generate bibliographies, outlines, and other research aids. Medical research can be made easier with ChatGPT’s ability to help form outlines and assist with literature reviews and data analysis. It can also help summarize relevant articles and identify key findings which would help medical researchers efficiently navigate the vast amount of information available on the internet.^8^

***5. Quick Access to information:*** ChatGPT can be utilized to provide accurate and up to date information on medical topics at a moment’s notice. This can include a range of topics from diseases, and their treatments to medical procedures. This can be useful for medical students and professionals who require quick access to information or clarification regarding a topic.^9^

***6. Generating Case Scenarios:*** ChatGPT can be used to generate case studies and scenarios to help medical students practice and improve their diagnostic and treatment planning abilities. This not only has the potential to help students develop their clinical reasoning skills but also prepare them for real-world clinical scenarios.

***7. Creating Content to Facilitate Learning***: Another function that ChatGPT provides is its ability to create content for summaries, quizzes, and flashcards. This can help educators create relevant, engaging, and interactive materials to help facilitate learning for their students.

***8. Language Translation:*** ChatGPT’s ability to translate language effectively can be utilized by medical professionals and educators to help communicate with patients from different linguistic backgrounds, in order to provide the best medical care.

However, it is imperative that we understand that although ChatGPT can be used for assistance in many fields of education, it is not a replacement for human teachers and educators and should not be thought of as such.

### ChatGPT Role in Clinical Management:

ChatGPT can be used in a clinical setting to manage patient data more easily. This can be done in several ways.^10^

***1. Documentation:*** It can be used to help generate clinical notes, summaries, and other documentation which would help optimize time and reduce the risk of human error.

***2. Decision Support:*** Although the final say of any medical decision should always be a health professional, ChatGPT may be used to help provide support and suggestions for treatment based on patient symptoms and medical history.

***3. Communication with patients:*** It may be used to generate automated responses to any queries the patients may have regarding appointment scheduling, as well as medication management.

### Limitations:

ChatGPT is an AI service which can generate text that is remarkably similar to human written content, but like any other statistical model it is not without errors. Its main limitations are deficiency of human-like understanding and lack of data input after 2021, which cause it to sometimes ignore the context of the prompt leading to generation of irrelevant text, or ideas and concepts that are not truly unique or original.^11^

Recently peer review papers were published with ChatGPT as an author.^12^ However, keeping in view the mentioned limitations, the World Association of Medical Editors (WAME) has recommended that ChatGPT should not be listed alongside the authors.^13^

## CONCLUSION

ChatGPT may be used as an assistance tool in medical education, research, and clinical management. However, it cannot be considered as a replacement for human capability and knowledge, as it is still plagued by the limitations that AI faces. However, we are witnessing a quantum leap in information technology, machine learning and AI. At this pace, it will be transforming our approach to medical education and clinical management in a matter of days. These changes should be seen and adopted with an open mind to make good use of them for improving medical education and clinical management.
